# HNP-1: From Structure to Application Thanks to Multifaceted Functions

**DOI:** 10.3390/microorganisms13020458

**Published:** 2025-02-19

**Authors:** Jiaqi Zhang, Zhaoke Liu, Zhihao Zhou, Zile Huang, Yifan Yang, Junzhu Wu, Yanhong Liu

**Affiliations:** 1 Department of Biochemistry and Molecular Biology, Center for Experimental Teaching of Basic Medical Science, School of Basic Medical Science, Wuhan University, Wuhan 430072, China; miniature2017@outlook.com (J.Z.);; 2 Department of Clinical Laboratory, Institute of Translational Medicine, Renmin Hospital of Wuhan University, Wuhan 430060, China

**Keywords:** human neutrophil peptide-1, defensins, antimicrobial peptide, drug resistance, biosynthesis

## Abstract

Antimicrobial peptides (AMPs) are critical components of innate immunity in animals and plants, exhibiting thrilling prospectives as alternatives to traditional antibiotics due to their ability to combat pathogens without leading to resistance. Among these, Human Neutrophil Peptide-1 (HNP-1), primarily produced by human neutrophils, exhibits broad-spectrum antimicrobial activity against bacteria and viruses. However, the clinical application of HNP-1 has been hampered by challenges associated with mass production and inconsistent understanding of its bactericidal mechanisms. This review explores the structure and function of HNP-1, discussing its gene expression, distribution, immune functions and the regulatory elements controlling its production, alongside insights into its antimicrobial mechanisms and potential clinical applications as an antimicrobial agent. Furthermore, the review highlights the biosynthesis of HNP-1 using microbial systems as a cost-effective alternative to human extraction and recent studies revealing HNP-1’s endogenous bactericidal mechanism. A comprehensive understanding of HNP-1’s working mechanisms and production methods will pave the way for its effective clinical utilization in combating antibiotic-resistant infections.

## 1. Introduction

**Figure 1:**
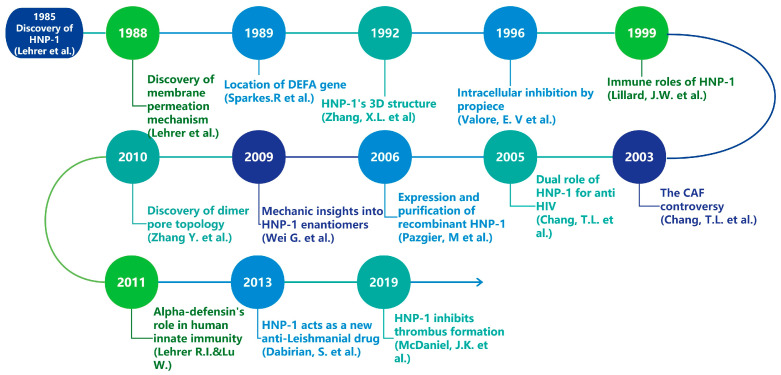
Timeline of major research discoveries related to HNP-1 [10,11,12,13,14,15,16,17,18,19,20,21,22].

Bacterial resistance represents a significant threat to public health in the 21st century [1,2]. Approximately 1.27 million deaths are attributed to bacterial resistance against existing antibiotics in 2019, and predictions suggest that this number will rise to 10 million in 2050 if alternatives are not found [3], which has intensified the urgency for researchers to discover novel antimicrobial agents including vaccines, AMPs, bacteriophages, probiotics, antivirulence and antibiofilm molecules, essential oils and others [4]. Defensins, small cationic peptides generally ranging from 2–5 kDa [5], are increasingly recognized as promising candidates for new antibiotics, forming a vital part of the antimicrobial peptide (AMP) repertoire [6]. In mammals, defensins display a characteristic β-sheet structure that is stabilized by three disulfide bonds and can be classified into three types: α, β, and θ, which differ in their distribution and the connectivity of six cysteine residues [7,8]. Furthermore, mammalian defensins are crucial components of both innate and adaptive immunity [7] and have garnered considerable interest due to their antibacterial properties. Human alpha defensins are subdivided into myeloid and enteric α-defensins based on their expression profiles and genetic organization [9]. Among these, α-defensin-1, also known as human neutrophil peptide-1 (HNP-1), is predominantly produced by human neutrophils and exhibits extensive antimicrobial activity. Since their initial discovery in 1985, defensins, including HNP-1, have captivated researchers’ interest due to their structural characteristics, applications, and production challenges (Figure 1) [10]. However, HNP1-related researches have never been specifically reviewed before. This review aims to provide a concise overview of HNP-1’s gene, structure, immunological effects, antimicrobial effects, and biosynthesis.

## 2. Gene, Structure and Distribution

Human neutrophil peptide 1 (HNP-1), along with HNP-2 and HNP-3, was first purified from azurophilic granules of human neutrophils in 1985 [10], and they comprise 30–50 percent of the total extractable myeloperoxidase (MPO) released by human polymorphonuclear neutrophils (PMNs) [23]. Since then, six alpha defensins have been identified in humans, five of which are densely located on chromosome 8p23.1. Although encoded by five different genes, human alpha defensins share a highly similar distribution pattern of disulfide bonds. HNP-1 to HNP-3 are encoded by two extremely similar genes: *DEFA1* and *DEFA3*, which exhibit significant copy number polymorphism related to immune functions and diseases [24,25,26].

**Figure 2:**
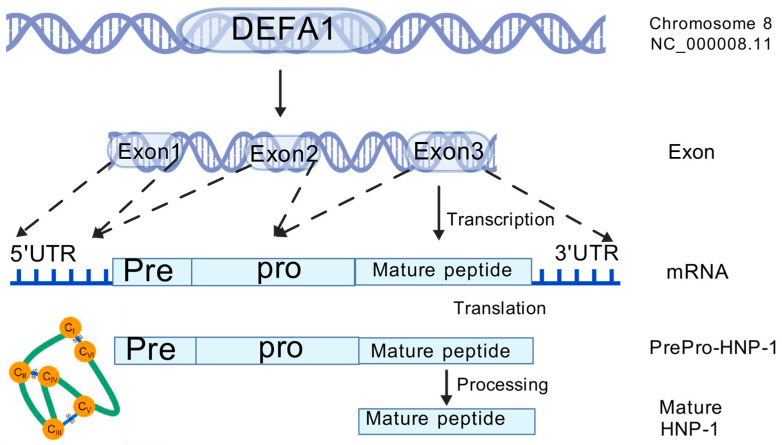
The genomic location, transcription, translation and posttranslational process of HNP-1.

**Figure 3:**
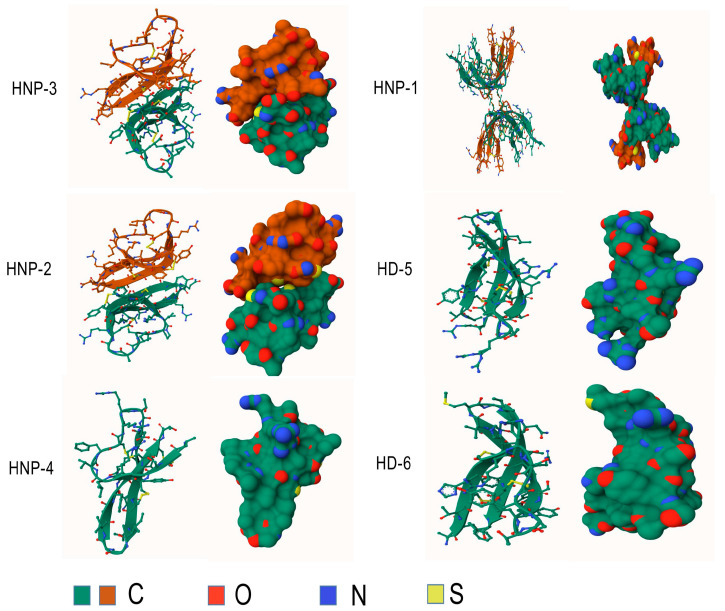
Structures of human alpha-defensins. Conserved disulfide bonds are shown in yellow.

Furthermore, their sequences exhibit considerable similarity [11], and the only difference is an amino acid residue at the N-terminus: Ala for HNP-1, Asp for HNP-3, and absence for HNP-2, which explains the absence of its own gene [12,27]. HNPs are translated as 94 amino acids (MRTLAILAAILLVALQAQAEPLQARADEVAAAPEQIAADIPEVVVSLAWDESLAPKHPGSRKNM**ACYCRIPACIAGERRYGTCIYQGRLWAFCC)** preproHNPs, which are co-translationally cleaved to 75 amino acids pro-peptides with a N-terminal prosegment having a negative charge that neutralizes the highly positively charged C terminal peptide. Further, 75 amino acids proHNPs are cleaved to a 56-amino acid intermediate form and onward to 29–30-amino acids mature HNPs [28] (Figure 2). Mature HNP-1, which comprises 30 amino acid residues, is enriched with cysteine (6; 20%), arginine (4; 13.3%), and alanine (4; 13.3%). The three-dimensional structure of HNP-1 was first elucidated by nuclear magnetic resonance spectroscopy (NMR) in 1992 [13,29]. Subsequently, the crystal structures of chemically synthesized HNP-1 and recombinant HNP-1 in a membrane-bound state were determined using solid-state NMR spectroscopy [14,30]. In the crystal structure, two HNP-1 monomers form a dimer through two pairs of intermolecular H-bonds (residues 18 and 20) between the two β2 strands, extending the triple-stranded β-sheet to a six-stranded β-sheet [31]. Structurally, six alpha defensins mutually exhibit three highly conserved disulfide bonds formed by six cysteines: C1:C6, C2:C4, C3:C5, which stabilize their three-stranded beta-sheet structure for dimerization, oligomerization, and multimerization, as well as protect them from degradation by processing enzymes [32,33] (Figure 3). In addition to disulfide bonds, they also share conserved Arg5, Glu13, and Gly17 (HNP1/HNP3 numbering). A salt bridge formed by Arg5 and Glu13 is important for stabilizing defensins from degradation by enzymes in vivo, but is irrespective of the folding and bactericidal activities of defensins [34]. Gly17 was discovered to assist in the formation of a classical beta bulge [35,36], and it also involves HNP-1’s dimerization and self-association, elucidating its conservation in all mammalian alpha-defensins [37]. Early in 1986, Daher et al. found that the inactivation ability of HNP-1 against herpes simplex virus 1 (HSV-1) is abrogated by the destruction of disulfide bonds through reduction and alkylation [38]. In the case of HNP-1, its disulfide bonds play a crucial role in almost all of its antimicrobial functions, including the direct inactivation of viruses and toxins, and binding to lipid II and pathogen proteins [39,40,41]. Notably, while linear HNP-1 is inactive, its cyclic analogs with different disulfide bond pairings do exhibit antimicrobial activity [42]. Moazzezy and coauthors revealed the truncated HNP-1 analogs’ elimination ability against multidrug-resistant *E. coli* and their anti-inflammatory activity in vivo [43,44]. Subsequently, they further designed modified peptides from HNP-1, which exhibited higher affinity for lipid II in silico, suggesting their potential antimicrobial activity [45].

Since the correct formation of HNP-1’s disulfide bonds is essential for its antimicrobial function, scientists have been working on in vitro folding for more than thirty years. One major challenge of chemical synthesis was the mismatch of cystine residues, so scientists applied orthogonal protecting groups to allow the sequential formation of correct disulfide bonds [31,42]. Due to orthogonal protection’s technically demanding and expensive nature, Lu and colleagues devised an optimized protocol using dimethylformamide (DMF), resulting in the effective folding of large quantities of highly pure HNPs [46]. Although HNP-1 is widely known as cationic, its function also depends on C-terminal hydrophobic residues, such as Trp26, which play an important role in binding pathogens, dimerization, and forming quaternary structures [47], therefore affecting its bactericidal and immunologic functions [31]. Mutational experiments on Tyr^16^, Ile^20^, Leu^25^, and Phe^28^ further illustrate that side chain hydrophobicity determines the activity at these sites.

HNP-1 has been detected in various tissues, including the placenta, spleen, thymus, intestinal mucosa, saliva, and cervical mucus plugs, highlighting their potential as disease biomarkers [48]. HNP-1 is reported to be especially highly expressed in a variety of cancers, including metastatic colorectal cancer, bladder cancer, renal cell carcinoma, squamous cell carcinoma, and breast cancer [49,50]. Also, HNP-1 exhibits anticancer activity in vitro as well as Lewis lung carcinoma cell tumor in mice [51,52]. Although HNPs are mainly produced by neutrophils, evidence does exist that *DEFA1* and *DEFA3* genes are upregulated in other immune cells during toxic epidermal necrolysis [53]. HNP-1 was also found in other cells, like lymphocytes [54] and NK cells [55], although we still do not know if they were indigenous or imported, since HNPs can be internalized in multiple ways [11], including the direct uptake of azurophilic granules [56], the pinocytosis of HNP-binding albumin or glycoprotein fetuin [57], and the ingestion of bacteria with HNPs adsorbed to their surface [58]. Interestingly, HNP-1 dimer is reported to bind to a low density lipoprotein receptor (LDLR), and further molecular docking suggests that the binding weakens bonds within the dimer, indicating a novel mechanism for internalization of HNP-1 in mammalian cells [59].

## 3. Synthesis and Release

HNP-1 is encoded as a 94-amino acid precursor protein, which contains three regions: pre, pro, and mature HNP-1, and its maturation requires a series of proteolytic processes to remove the pre and pro regions [28]. The pre-region (19 amino acids) is a hydrophobic (N)-terminal signal sequence that inserts into the endoplasmic reticulum to direct HNP-1 to granules [60]. The subsequent pro-region (45 amino acids) acts as a molecular chaperone for mature HNP-1 (30 amino acids), which is vital for its correct folding. Additionally, the pro-region temporarily neutralizes mature HNP-1’s positive charge to prevent intracellular reactions before release [15,61]. HNP-1 is primarily produced in the granules of immature neutrophils and other immune cells [62]. It is synthesized constitutively by the bone marrow precursors of neutrophils during specific differentiation stages of neutrophil development, particularly in promyelocytes and early myelocytes [63]. Although neutrophils contain a large amount of HNP-1, once they mature, they no longer synthesize HNP-1’s mRNA or the peptide. HNP-1, together with HNP-2 and HNP-3, is present in primary azurophil granules at high concentrations, and most of them are directed to fuse with phagocytic vacuoles to directly kill phagocytosed pathogens [64,65]. Upon inflammation, the remaining granules containing HNP-1 are released near the pathogen surface during holocrine secretion as neutrophils begin to degranulate. The binding pattern of HNP-1 is mostly non-specific, with the exception of its affinity for anionic molecules and carbohydrates. Additionally, many theta and alpha defensins share a lectin-like affinity for glycosylated molecules, making many anionic, glycosylated cell surfaces highly attractive to them. However, these bindings are non-covalent and reversible; therefore, they can easily relocate onto another nearby virus or bacterium [66,67]. The PMNs carrying HNP-1 follow the granulocyte positioning system, which directs them to invading pathogens. Armed with cationic properties, they kill pathogens directly by penetrating cell membranes [28,68]. Although the human alpha defensin family has diverged dramatically during evolution, their promoter regions exhibit significant conservation compared to the highly variable non-promoter regions in HD5 and HD6 [69]. The conserved promoter region of HNPs shares multiple regulatory elements related to the maturation of the HL-60 myeloid cell line, illustrating HNP-1’s function as a maturation marker [70]. The promoter regions of *HDEFA1/1A* contain binding sites for the myeloid factor PU.1 [70] and C/EBP-alpha [71] that control its constitutive transcription in promyelocytes. HNP-1’s expression can also be modulated by factors such as granulocyte colony-stimulating factor (G-CSF) [72].

## 4. Immune Functions

Apart from its attractive antimicrobial activity, HNP-1 also plays crucial roles in innate and adaptive immunity, including chemotaxis, phagocytosis, and cytokine induction (Table 1). In vitro, HNP-1 exhibits significant chemotactic activity for human monocytes, compared to HNP-2 and HNP-3, which show less and no chemotactic activity, respectively [73]. HNP-1 also forms heteromers with platelet-derived C-C motif chemokine receptor 5 (CCR5), which stimulates monocyte adhesion through CCR5 ligation, and disrupting the formation of this heteromer results in attenuation of monocyte and macrophage recruitment [74,75]. Moreover, HNP-1 acts as a chemotactic agent for macrophages, human CD4+/CD45RA+ naive T cells, human CD8+ T cells, immature human dendritic cells and human mast cells like HMC-1 [76]. In addition to monocytes, HNP-1 selectively induces the migration of human CD4+/CD45RA+ naive and CD8+ T cells, but not CD4+/CD45RO+ memory T cells. It is also chemotactic for immature human dendritic cells and murine dendritic cells, suggesting a non-specific binding mechanism to receptors [77]. In the context of phagocytosis, HNPs were reported to aggregate pathogens to promote their intake [78]. With regards to cytokine induction, HNP-1’s effect is like a double-edged sword. A low concentration of HNP-1 upregulated the expression of TNF-alpha and IL-1 beta while downregulating IL-10 in monocytes activated by *S. aureus* or phorbol12-myristate13-acetate (PMA) in vivo, which does not exist in vitro. Conversely, high concentrations of HNP-1 exhibit cytotoxic effects on monocytes in serum-free medium, although its cytotoxicity is abrogated in the presence of serum and blood, indicating an in vivo mechanism [79]. Considering the complexity of its cytokine-induction effect, we further elucidate from two aspects: pro-inflammatory and anti-inflammatory.

### 4.1. Inflammatory Effects

In the presence of inflammation, HNPs and self-DNA form neutrophil extracellular traps (NETs), which promote plasmacytoid dendritic cells (pDC) activation [80]. In acquired host defense responses, HNP-1 enhances systemic IgG antibody responses in mice. This effect was achieved through the induction of antigen-specific CD4+ T cell proliferative responses and IFN-γ, IL-5, IL-6, and IL-10 secretion [16]. Structural research further reveals that HNP-1 selectively inhibits the Kv1.3 channel and the secretion of IL-2 in human CD3(+) T cells [81]. Interestingly, in acquired thrombotic thrombocytopenic purpura (TTP), HNP-1 to -3 are reported to bind the central A2 domain of von Willebrand factor (VWF), thus blocking ADAMTS13 binding and providing a novel link between inflammation/infection and the onset of microvascular thrombosis [82,83,84]. HNP-1 was also found to induce the release of IL-1 beta and activate the NLRP3 inflammasome through binding to P2X7 in LPS-primed macrophage cell lines [85]. HNP-1’s induction of IL-8 in human epithelial cells was related to P2Y6, purinergic P2 receptors and G-protein coupled nucleotide receptor P2Y6 signaling pathway [86,87]. Further comparison studies showed that the activation of ERK1/2 and PI3K/Akt pathways is shared by monocytes and lung epithelial cells, but monocytes specifically depend on Src kinase [88].

### 4.2. Anti-Inflammatory Effects

In addition to inducing immune responses, HNP-1 is also involved in anti-inflammatory activities. HNP-1 inhibits both the classical and the lectin pathways of complement activation through interaction with C1q and MBL, indicating its role in protecting tissues from inflammation [89]. HNP-1 significantly inhibits spontaneous and cytokine-inducible NK cell activity, as well as the production of IFN-gamma and IL-6 by peripheral blood mononuclear cell (PBMCs) [90]. Furthermore, human PMNs secrete HNP-1 to inhibit the migration of other PMNs in response to stimuli [91]. HNP-1 inhibits TNF-alpha produced by human monocyte-derived macrophages (HMDMs), as well as the activation of both T cell-mediated and LPS-mediated macrophages, the production of pro-inflammatory cytokines and release of IL-1 beta from LPS-activated monocytes [92]. Overall, HNP-1, in a case with necrotic macrophages, protects mice from experimental inflammation [93]. Interestingly, HNP-1 exhibits a tertiary structure-dependent inhibition of bulk mRNA translation within HMDMs without interfering with mRNA transcription or stability, preventing excessive pro-inflammatory responses [68]. HNP-1, along with other Paneth cell defensins, blocks the release of IL-1 beta induced by ATP or PG-1 from LPS-activated monocytes and inhibits the synthesis of pro-IL-beta proteins, suggesting its role in a signaling step shared by both mechanisms.

**Table 1:** HNP-1’s immune functions and targets.

Function	Target Cell	Effect/Signaling	Reference
cytokine induction	LPS-activated monocytes monocytes activated by *S. aureus* or PMA mononuclear cell lines airway epithelial cells	blocked the release of IL-1 beta upregulated the expression of TNF-alpha and IL-1beta while downregulating IL-10 release of IL-1 beta upregulate the synthesis of IL-8	[92] [92] [92] [79]
chemotactic effect	human monocytes platelet human mast cells macrophages CD4+/CD45RA+ naive CD8+ T cells immature human dendritic cells immature murine dendritic cells	adhesion CCR5 chemotactic agent chemotactic agent migration migration TNF-alpha TNF-alpha	[71] [72,73] [74] [74] [81] [81] [81] [82]
tumor cell lysis	murine teratocarcinoma	abrogates its oncogenicity in vivo	[94]
	mouse 4T1 breast cancer	increased the tumor’s susceptibility to doxorubicin (Dox)	[95]
	A549 lung cancer	increased the tumor’s susceptibility to doxorubicin (Dox)	[95]

### 4.3. Anticancer Effects

Concerning tumor cells, HNP-1 was found to exhibit concentration-dependent tumor cell lysis activity; in vitro exposure to HNP-1, 2, and 3 of murine teratocarcinoma abrogates its oncogenicity in vivo [94]. In a mouse 4T1 breast cancer model mimicking locally advanced breast cancer (LABC), intratumoral injection of plasmid HNP-1 increased the tumor’s susceptibility to doxorubicin (Dox), and this effect was also observed in an A549 lung cancer model treated with cisplatin (DDP) and pHNP-1 [95]. Atomic force microscopy further reveals that HNP-1 tends to bind solid tumor cells compared to human leukemia cells, and it could induce apoptosis through cellular membrane disruption at low concentrations [96]. Preclinical safety investigations of HNP-1 gene therapy on tumors conducted in nonhuman primates also support future clinical studies of pHNP-1-based local gene delivery in tumor patients [97].

## 5. Antimicrobial Functions and Underlying Mechanisms

When it comes to HNP-1’s ability against pathogens, two practical considerations are the actual concentration of HNP-1 in plasma and the effects of pH and other components in its working environment. While the theoretical concentration of HNP1-3 is impressive, with human PMNs carrying 15–30 µg of HNP-1 in each milliliter of normal blood, the actual concentration of HNP1-3 is below 100 ng/mL, which means that only a small part of HNP-1 could function normally, leading us to reconsider its activity in vivo since concentration plays a vital role in its multifaceted functions [98]. This disparity arises for two reasons: neutrophils only release a relatively small amount of HNPs into the plasma, and HNP-1’s affinity for glycoproteins, which are abundant on cell surfaces. Even if HNP-1 aggregates for some reason, such as in the gingival crevice [99], an excessively high concentration could actually convert its defensive effect into immunological damage [100]. Additionally, apart from glycoproteins, HNP-1 tends to bind serum proteins when its concentration rises during sepsis [101]. Since HNP-1 exhibits different modes of action at varying concentration levels, determining its actual concentration in the working environment is essential for elucidating its antimicrobial mechanism [102].

### 5.1. Anti-Viral Effects

#### 5.1.1. Anti-HIV Activity

HNP-1 is renowned for its antiviral activity, particularly against human immunodeficiency virus (HIV) [17]. Its anti-HIV activity was first discovered in 2002 by Cohen et al., who did not recognize it and referred to it as a “mystery factor” [103]. In the same year, HNP1-3 was identified as components of the CD8 T cell’s anti-HIV-1 factor. However, Chang and colleagues later stated that HNP-1’s HIV inhibition differs from that of the CD8(+) T-lymphocyte antiviral factor (CAF) [18]. In 2005, the same group systematically studied HNP-1’s inhibition mechanism and found that HNP-1 inhibited PKC phosphorylation, thereby blocking HIV’s nuclear import and transcription. HNP-1 and HBD-2 inhibited HIV-1 replication even when added 12 h post-infection and blocked viral replication following HIV-1 cDNA formation; they also downmodulated CXCR4. Moreover, RTD-1 inactivated X4 HIV-1, while HNP-1 and HBD-2 inactivated both X4 and R5 HIV-1 [104]. HNP-1 was found to inhibit multiple steps of HIV entry, including: (i) Env binding to CD4 and coreceptors; (ii) refolding of Env into the final six-helix bundle structure; and (iii) productive HIV-1 uptake, but not internalization of endocytic markers [105]. HNP-1 contributed to the HIV neutralization ability of cervicovaginal secretions from women in HIV-serodiscordant relationships [106]. Sub-inhibitory doses of HNP-1 significantly prolonged the lifetime of gp41 intermediates, thereby enhancing the activity of several anti-gp41 antibodies and peptide inhibitors [107]. Interestingly, while HNP-1 does exhibit anti-HIV activity in vitro, there is evidence that elevated levels of HNP-1 correlate with increased HIV acquisition [108]. Therefore, Valere et al. further investigated and found that HNP-1 increased epithelial permeability, which paralleled increased HIV transmission, indicating that HNP-1 indirectly facilitates the invasion of HIV [109].

#### 5.1.2. Other Viruses

HNP-1’s antiviral effect in vitro was first elucidated in the 1980s when researchers found that HNP-1 could directly inactivate HSV-1, HSV-2, cytomegalovirus, vesicular stomatitis virus, and influenza virus A/WSN, while non-enveloped viruses, including echovirus type 11 and reovirus type 3, are resistant to direct inactivation [110]. However, later research revealed that HNP-1’s direct inactivation is largely abolished by the presence of serum or albumin, and removal of HNP-1 before adding the virus still protects cells from infection, suggesting the existence of a cellular signaling pathway. In vivo, HNP-1, -2, and -3, as well as HD5, have been reported to exist in the female genital tract at levels exceeding those that inhibit human papilloma virus (HPV) in vitro, with further experiments revealing that they are potent antagonists of infection by both cutaneous and mucosal papillomavirus types [38]. Beyond HIV, HNP-1’s inhibition of PKC phosphorylation also plays a role in inhibiting Influenza A virus (IAV) [111], human adenovirus serotype 3 [112], and adenovirus type 35 [113]. HNP-1 also exhibits lectin-like properties that promote IAV agglutination, thereby increasing PMN uptake of the virus [114]. Thermodynamic studies reveal that HNP-1 induced destabilization leads to exposure of hydrophobic groups in viral proteins [115]. Later, it was demonstrated that HNP-1, -2, and -3 blocked adenovirus uncoating during cell entry using single-cell analysis. HNP-1’s antiviral activity was also revealed in viral hemorrhagic septicemia rhabdovirus (VHSV), and it was reported to elicit upregulation of IFN [116]. For SARS-CoV-2, HNP-1 is reported to show protective effects by inhibiting viral fusion and binding to the SARS-CoV-2 Spike protein [117,118].

### 5.2. Antibacterial Effects

Due to the complex functions that human PMNs possess, it is challenging to illustrate the exact antibacterial mechanism of HNP-1 in vivo. Although scientists performed a knock-in experiment on murine RAW 264.7 macrophages and found that HNP-1 expression in RAW cells effectively restrained the intracellular growth of Histoplasma capsulatum, additional evidence in humans is still required to identify its actual functions in vivo [119]. The antibacterial mechanisms of HNP-1 in vitro were first elucidated in 1989 [120]. However, as the structure-function relationships of HNP-1 continued to be uncovered, controversy regarding the differences in its bactericidal mechanisms against Gram-negative and Gram-positive bacteria began to emerge (Table 2).

In Gram-negative bacteria, HNP-1 sequentially permeabilizes the outer and inner membranes, and the inhibition of DNA, RNA, and protein synthesis parallels the breakage of the inner membrane [19,120]. Xie et al. revealed that HNP-1 interferes with the DNA damage response pathway by inhibiting RecA’s binding to ssDNA [121]. Additionally, *Escherichia coli* may survive HNP-1 exposure once its growth is inhibited or its transmembrane potential is eliminated [122]. Further investigation of bilayer membranes found that HNP-1 is able to form channels immediately, and this activity relies on disulfide bonds and the membrane potential [123]. Later, solid-state NMR studies revealed HNP-1’s oligomerization tendency and its ’dimer pore’ topology, in which the polar top of the dimer lines an aqueous pore while the hydrophobic bottom faces the lipid chains [124]. However, the significance of dimerization in HNP-1’s bactericidal activity is selective: impairing dimerization ability has a more substantial effect on killing *Staphylococcus aureus* than *Escherichia coli*. In *Salmonella typhimurium*, It was later identified that its resistance to defensins (HNP-1 and HNP-2) was associated with both PhoP and PhoQ, suggesting that a phoP-activated gene (pag) was responsible for defensin resistance. Further structural investigation revealed that the anionic periplasmic domain of Salmonella PhoQ was necessary for the induction of HNP-1 and HNP-2, and this effect persists in the presence of physiological Mg2+ concentrations but can be restored at higher concentrations due to competitive binding [31,124,125]. In *Klebsiella pneumoniae*, HNP-1 decreases LPS, CPS content, and outer membrane proteins of *K. pneumoniae*, promoting phagocytosis to accelerate bacterial elimination [126]. In *Streptococcus pyogenes*, sublethal concentrations of HNP-1 preferentially target the ExPortal, a unique microdomain for protein secretion and processing [127]. Conversely, Gram-negative bacteria also exhibit anti-HNP-1 capabilities: cholera toxin (CT) from *Vibrio cholerae* and heat-labile enterotoxin (LT) from *Escherichia coli* both modify HNP-1, while *Neisseria meningitidis* NarE’s transferase activity is significantly inhibited by HNP-1 [128]. In Gram-negative enteric bacteria, envC has been identified as a conserved CAMP resistance factor [129]. Kudryashova et al. investigated the mechanism of HNP-1’s inactivation of bacterial protein toxins and found that HNP-1 unfolded the toxin’s thermodynamically unstable region [130]. In *Fusobacterium nucleatum*, HNP-1 resistance arises due to inhibition of membrane permeability, along with increased planktonic and established biofilm growth [131]. Transmission electron microscopy (TEM) analysis revealed a decrease in the outer membrane and the formation of rough vesicles attached to the outer membrane, suggesting a structural adaptation to HNP-1 exposure [132]. In *Pseudomonas aeruginosa*, HNP-1 binds to and directly inactivates its virulence factor, exotoxin A (ETA) [133]. Interestingly, HNP-1 also acts as a facilitating factor for *Shigella* adhesion and invasion by binding to its outer membrane through hydrophobic residues [134].

In killing Gram-positive bacteria, however, HNP-1 proved to be more potent than human beta defensins (HBDs), despite HBDs being significantly more cationic, suggesting a cationicity-independent mechanism distinct from that of Gram-negative bacteria. Furthermore, HNP-1’s antibacterial activity against *S. aureus* shows little association with its membranolytic activity [135]. Mutational experiments suggest a strong relationship between residue 26 (tryptophan in HNP-1) and its ability to target *Staphylococcus aureus*, as well as its capacity for dimerization and oligomerization, indicating that hydrophobicity may play a role in killing *S. aureus*. More recently, researchers discovered that HNP-1 targets lipid II, a precursor of bacterial cell walls, suggesting that HNP-1 may inhibit the synthesis of lipid II. Based on the HNP-1-lipid II complex, low molecular weight compounds that mimic this interaction were chemically identified, proving protective in an in vivo model for sepsis [30]. Further investigations into HNP-1 enantiomers found that the L-enantiomer’s bactericidal activity against *S. aureus* significantly exceeds that of the D-enantiomer, and the D-enantiomer’s lower affinity for lipid II is consistent with this suggestion [20]. Interestingly, this mechanism is also shared by other antimicrobial peptides (AMPs) including HBD-3, indicating a mutual mechanism against Gram-positive bacteria [136]. Peschel et al. identified a virulence factor, MprF, which led to the reduction of the negative charge on the cell surface, thereby decreasing the binding ability of HNP-1 to bacteria. Intriguingly, MprF-related genes were also identified in several pathogens, including *Mycobacterium tuberculosis*, *Pseudomonas aeruginosa*, and *Enterococcus faecalis*, suggesting the existence of a common AMP-resistant mechanism [137,138]. Collins et al. revealed that the dlt operon is a determinant of *S. aureus* resistance to HNP-1, -2, and -3, mediating d-alanine incorporation into teichoic acids in the staphylococcal cell envelope [139]. Later, Yang et al. found that the expression of mprF and dlt, which determine the positive net charge on the cell surface, is dependent on the co-transcription of both graR and vraG; however, HNP-1 alone could not induce the transcription of mprF and dlt via the graRS-vraFG pathway [140]. Further investigation reveals that cationic peptides directly interact with extracellular loops to activate GraS [141]. Jin et al. demonstrated that staphylokinase could induce the release of HNP-1 and HNP-2 from polymorphonuclear cells, and it also inactivates HNPs by forming complexes with them, thus neutralizing HNPs’ bactericidal effect in vivo [142]. HNP-1 could also neutralize *S. aureus*’s Panton-Valentine leukocidin (PVL) by binding to both subunits of the toxin [143].

Apart from viruses and bacteria, HNP-1’s interactions with parasites and fungi have also been studied. For *Leishmania* spp., HNP-1 treatment on neutrophils significantly delays the onset of apoptosis, and the infectivity of Leishmania into neutrophils in vitro is significantly reduced [21]. The remedial effects of HNP-1 on *Leishmania* spp. are also promising; it effectively induces Th1 polarization and restricts parasite burden, thereby controlling disease progression [144]. In *Candida albicans*, Salvatori et al. found that the deletion of its ras gene increased its resistance to HNP-1’s non-oxidative killing due to heightened catalase activity [145].

**Table 2:** HNP-1’s bactericidal effects and mechanisms.

Bacteria	Bactericidal Mechanism	Resistant Mechanism	Reference
*Histoplasma capsulatum*	HNP-1 restricts intracellular growth	-	[114]
*Escherichia coli*	Permeabilizes membranes	-	[115,116]
*Escherichia coli*	Inhibits DNA/RNA/protein synthesis		[118]
*Salmonella typhimurium*	Forms channels in membranes	PhoP and PhoQ dependent defensin resistance	[121,124]
*Klebsiella pneumoniae*	Reduces LPS and outer membrane proteins	-	[124]
*Staphylococcus aureus*	Targets lipid II; disrupts membrane	Dlt operon reduces HNP-1 binding	[136,139]
*Streptococcus pyogenes*	Targets ExPortal for secretion	-	[125]
*Vibrio cholerae*	-	Modifies HNP-1	[126]
*Fusobacterium nucleatum*	-	Inhibition of membrane permeability	[129,130]
*Pseudomonas aeruginosa*	Inactivates exotoxin A	-	[131]
*Shigella*	Facilitates adhesion and invasion	-	[132]
*Mycobacterium tuberculosis*	-	MprF reduces negative charge on surface	[137,138]
*Enterococcus faecalis*	-	MprF-related resistance	[137]

## 6. Biosynthesis and Mass Production

Due to the modest concentration of HNP-1 in plasma, extracting and purifying mature HNP-1 directly from human blood is both costly and technically demanding. Existing methods of chemical synthesis also encounter challenges related to high costs and stringent conditions, as well as complex procedures, since most of HNP-1’s biological functions require its correct folding. Consequently, scientists have been working on generating mature HNP-1 using engineered bacteria and mammalian cells [22,146]. Although mammalian cells produce significantly more proteins than bacteria, purifying HNP-1 from these cells has proven to be difficult and expensive, despite some progress in producing HNP-1 using modified sheep mammary gland cells [147]. Additionally, Zhang et al. successfully expressed recombinant HNP-1 in *Pichia pastoris* with an upstream cleavage site, which allowed for the production of correctly folded HNP-1, and its functional analysis indicated potent antibacterial activity against drug-resistant *Helicobacter pylori* [148]. Due to HNP-1’s bactericidal activity, the yield of recombinant HNP-1 in engineered bacteria is seriously restricted. Xie et al. found that mature HNP-1 is activated during the expression of full-length HNP-1, triggering bacterial programmed cell death [121]. In comparison, endogenous HNP-1 kills bacteria at a much lower concentration than exogenous HNP-1, suggesting a different bactericidal mechanism than the cationic penetration hypothesis. However, the processing and bactericidal mechanisms of endogenous HNP-1 remain to be elucidated, posing challenges for the manufacture of HNP-1-resistant bacteria.

Endogenous full-length HNP-1 is initially encoded as a 94-amino acid pre-pro-HNP-1, which includes a 19-amino acid hydrophobic signal peptide (pre-region), a 45-amino acid anionic pro-region, and a 30-amino acid cationic peptide at the C-terminus [149]. It has been established that endogenous HNP-1 can be transported to the periplasm, a process that occurs through three known pathways: the general secretory pathway (Sec), the signal-recognition particle (SRP) pathway, and twin-arginine translocation (Tat) [150,151]. By analyzing the signal peptide sequence of HNP-1, we found that it resembles the recognition sequences of both the Sec and SRP pathways. Further identification of protein–protein interactions using His-tag and gene ontology enrichment analysis suggested that pre-pro-HNP-1 likely interacts with multiple periplasmic secretion proteins (SecY, YegQ, YcbZ, FFH, FtsY, SecA, PrlC, YdcP, LepB), electron transport chain proteins (SdhB, YkgF, CyoB, CyoA, CydB, NuoF, NuoC, NuoG, NuoA, UbiG), and LPS-related proteins (LptD, LpxT, WzzE, LptB, LpxA, LptG) (Supplementary Materials). The Sec translocation pathway is the most frequently used post-translational translocation pathway in *E. coli*, meaning that translated peptides are guided through membranes in an unfolded or partially folded state. The Sec pathway involves seven translocation proteins: SecA, SecB, SecD, SecF, SecY, SecE, and SecG, of which SecY, SecE, and SecG are shared with the SRP pathway [152]. The SRP is a co-translational translocation pathway, meaning that once the signal peptide is translated by the ribosome, the protein SRP and FtsY bind to it, forming a ribosome-peptide-SRP complex [153]. The peptide continues to be translated as the complex guides it to the SecYEG transmembrane channel. The protein–protein interaction between FtsY and pre-pro-HNP-1 has been primarily confirmed, however, further research remains necessary to investigate other related proteins.

Regarding the endogenous bactericidal mechanism, two approaches are currently considered: ROS-mediated apoptotic-like death (ALD) and inhibition of LPS transportation [154]. *E. coli* has two known programmed death mechanisms: apoptotic-like death and mazEF-mediated death. After inducing HNP-1 expression, we observed that ALD-related Edin genes were significantly upregulated compared to the mazE and mazF genes, thereby confirming that HNP-1 induced programmed cell death through ALD [121]. Concurrently, the protein–protein interactions between pre-pro-HNP-1 and electron transport chain proteins suggest that HNP-1 may disrupt respiratory processes, resulting in the generation of high levels of ROS and the activation of ALD. LPS is an essential component of *E. coli*’s outer membrane, synthesized inside the bacteria. It must be transported from the inner to the outer membrane by the LptB2FGC complex in the periplasm. Zampaloni and Pahil reported an antibiotic called Zosurabalpin, which inhibits LPS transportation and kills bacteria through the accumulation of LPS within and the rupture of the outer membrane [155,156]. Since pre-pro-HNP-1 exhibits interactions with multiple LPS-related proteins, it would be valuable to further investigate whether mature HNP-1 and pro-HNP-1 interact with LPS-related proteins.

Following translocation, pro-HNP-1 is cleaved in the periplasm to remove the pro-region, allowing the maturation of mature HNP-1 [157]. Since pre-pro-HNP-1 and pro-HNP-1 exhibit no toxicity when exogenously present, it may be promising to inhibit the splicing of the pre- or pro-regions in bacteria before purifying and splicing them artificially to acquire mature HNP-1. Tongaonkar et al. identified neutrophil elastase (NE) and proteinase 3 from azurophil granules as the pro-HNP-1 convertases that activate correctly folded pro-HNP-1 and extensively degrade misfolded HNP-1 conformers [157]. However, the exact splicing enzyme in *E. coli* is still unknown, although several suspected enzymes have been identified in unpublished data. Therefore, it will be important to further investigate whether these enzymes actually exist and if they are involved in the splicing of HNP-1.

## 7. Future Perspectives

Firstly, as we elucidated above, while the mass production of proteins using bacteria has achieved success for many proteins, HNP-1, along with many other antimicrobial peptides (AMPs), faces a dilemma. On one hand, engineered bacteria can indeed produce correctly folded HNP-1; on the other hand, the increase in the product’s yield and activity shortens the bacteria’s survival time, thereby restricting the overall yield of biosynthesis. To combat this challenge, it’s a good idea to inhibit HNP-1’s toxicity is to prevent its cleavage inside bacteria as pre-pro-HNP1 and pro-HNP1 is not toxic to bacteria. However, as we mentioned above, little is known about the exact cleaving enzyme of immature HNP-1, posting a tough challenge. Another approach is identifying more target proteins of HNP-1, as modification of these targets may prolong engineered bacteria’s survival time against endogenous HNP-1, thus improving the overall yield. Notably, our previous work suggested that HNP-1 was probably translocated through Sec and SRP pathways, and its endogenous bactericidal activity is likely related to ALD and inhibition of LPS transportation (elaborated above). Therefore, identification of related proteins might be a promising direction.

Secondly, as a short peptide comprised of only thirty amino acids, the majority of structural studies on HNP-1 were completed over a decade ago. However, in recent years, the rapid advancements in structural biology and computational biology have led to the development of numerous protein design and protein interaction prediction tools including RoseTTA fold, ProteinMPNN as well as RFdiffusion, providing new opportunities to further explore the mechanisms of interaction between HNP-1 and target proteins, receptors, and other entities [158,159,160]. Researchers have already begun to design, computationally simulate, and synthetically produce HNP-1 analogues based on this approach [43,44,45]. Additionally, the direct application of natural HNP-1 poses a risk of enhancing the resistance of pathogenic microorganisms to human innate immune peptides. Therefore, de novo design and synthesis of proteins based on the structure of HNP-1 and its homologous proteins may facilitate the development of antimicrobial drugs that are not only more readily synthesized but also more efficient than their natural counterparts.

## 8. Conclusions

As challenges posed by drug-resistant pathogens continue to emerge, the search for novel antibiotics has become vital and pressing. HNP-1, as a vital component of human innate immunity, offers a promising solution for addressing the drug-resistant crisis. Despite promising antimicrobial and immunological properties of HNP-1, its clinical application has been hampered by restrictions in its synthesis and insufficient understanding of its mechanism of action. Also, inconsistent mechanisms of action in vivo and in vitro have limited HNP-1’s use. Therefore, it would be meaningful to further investigate HNP-1’s exogenous and endogenous bactericidal mechanisms, as well as its multifaceted immunological functions, paving the way for strategies for its mass production and clinical application.

## Data Availability

No new data were created.

## Supplementary Materials

The following supporting information could be downloaded from https://doi.org/10.1128/spectrum.00860-21 (accessed on 26 September 2024). Supplementary Materials mentioned above come from the work our group done in 2022 (Xie, Q.; Wang, Y.; Zhang, M.; Wu, S.; Wei, W.; Xiao, W.; Wang, Y.; Zhao, J.; Liu, N.; Jin, Y.; et al. Recombinant HNP-1 Produced by Escherichia Coli Triggers Bacterial Apoptosis and Exhibits Antibacterial Activity against Drug-Resistant Bacteria. Microbiol. Spectr. 2022, 10, e00860-21. https://doi.org/10.1128/spectrum.00860-21.), related data could be accessed from Supplementary Materials of this paper.
